# Use of Infrared Thermography and Heart Rate Variability to Evaluate Autonomic Activity in Domestic Animals

**DOI:** 10.3390/ani14091366

**Published:** 2024-05-01

**Authors:** Marcelo Daniel Ghezzi, María Carolina Ceriani, Adriana Domínguez-Oliva, Pamela Anahí Lendez, Adriana Olmos-Hernández, Alejandro Casas-Alvarado, Ismael Hernández-Avalos

**Affiliations:** 1 Anatomy Area, Faculty of Veterinary Sciences (FCV), Universidad Nacional del Centro de la Provincia de Buenos Aires (UNCPBA), University Campus, Tandil 7000, Argentina; 2 Centro de Investigación Veterinaria de Tandil CIVETAN, UNCPBA-CICPBA-CONICET (UNCPBA), University Campus, Tandil 7000, Argentina; 3 Neurophysiology, Behavior and Animal Welfare Assessment, DPAA, Universidad Autónoma Metropolitana, Xochimilco Campus, Mexico City 04960, Mexico; 4 Division of Biotechnology—Bioterio and Experimental Surgery, Instituto Nacional de Rehabilitación-Luis Guillermo Ibarra Ibarra (INR-LGII), Tlalpan, Mexico City 14389, Mexico; 5 Clinical Pharmacology and Veterinary Anesthesia, Facultad de Estudios Superiores Cuautitlán, Universidad Nacional Autónoma de México (UNAM), Cuautitlán Izcalli 54714, Mexico

**Keywords:** autonomic nervous system, infrared thermography, skin blood flow, surface temperature, stress-induced hyperthermia

## Abstract

**Simple Summary:**

The following review is based on the use of infrared thermography as a method to assess stress-related autonomic activity and its association with cardiovascular and heart rate variability in domestic animals. Stress-induced hyperthermia is one of the main physiological responses of animals to a stressor. The increase in body temperature activates compensatory peripheral mechanisms to increase heat dissipation and prevent further temperature increases. Changes in skin blood flow cause alterations in cutaneous temperature, alterations that can be assessed through infrared thermography and that will be discussed in the present review.

**Abstract:**

Most of the responses present in animals when exposed to stressors are mediated by the autonomic nervous system. The sympathetic nervous system, known as the one responsible for the “fight or flight” reaction, triggers cardiovascular changes such as tachycardia or vasomotor alterations to restore homeostasis. Increase in body temperature in stressed animals also activates peripheral compensatory mechanisms such as cutaneous vasodilation to increase heat exchange. Since changes in skin blood flow influence the amount of heat dissipation, infrared thermography is suggested as a tool that can detect said changes. The present review aims to analyze the application of infrared thermography as a method to assess stress-related autonomic activity, and their association with the cardiovascular and heart rate variability in domestic animals.

## 1. Introduction

The autonomic stress response to events that challenge the homeostasis of animals—including distress, diseases, or pain—is modulated by both branches of the autonomic nervous system (ANS): the sympathetic (SNS) and parasympathetic nervous system (PNS) [1,2]. The coordination of the central and peripheral responses to a stressor involves the hypothalamic–pituitary–adrenal (HPA) and the sympathetic–adrenomedular (SAM) axes [3]. These systems activate physiological, endocrine, and metabolic adaptive mechanisms to ensure the animal’s survival [4]. Among these changes, modifications in the skin’s sympathetic blood flow can occur due to physical, mental, and thermal stress [5,6,7], which can be non-invasively evaluated through infrared thermography (IRT) [8,9]. 

The activation of the SNS during the perception of a stressor leads to cardiovascular changes such as increasing heart rate (HR), vascular resistance, and body temperature mainly by the action of catecholamines (e.g., norepinephrine (NE)) released from the adrenal medulla as part of the “fight or flight” reaction [2,10]. Stress-induced hyperthermia (SIH) is a phenomenon where body temperature increases due to cardiovascular changes (tachycardia) inducing thermogenesis [11]. Increases in core temperature also activate vasodilator mechanisms to increase heat exchange in skin blood vessels [12]. Since IRT is a tool that can detect changes in the surface temperature of animals, it can indirectly assess sympathetic-mediated vasomotor changes [13,14,15,16]. This article aims to analyze the application of IRT as a method to assess stress-related autonomic activity, and their association with cardiovascular and heart rate variability in domestic animals.

## 2. Importance of Evaluating the Activity of the Sympathetic/Parasympathetic Pathways

**Figure 1:**
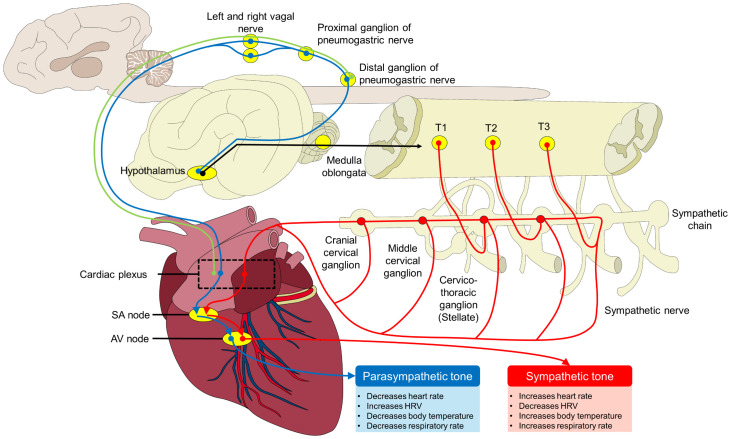
Schematic representation of the autonomic control of the cardiovascular function. Parasympathetic (blue lines) and sympathetic nerves (red lines) control the responses of animals to stress. Sympathetic-mediated responses involving the cardiorespiratory system (e.g., tachycardia, tachypnea, or hyperthermia) are triggered by the action of pre- and post-ganglionic nerves (black lines) and the projection of the stimulus through the sympathetic chain. In contrast, parasympathetic-related responses, mediated by cardiac ganglia neurons, include decreases in heart rate, body temperature, and respiratory rate, and increases in heart rate variability (HRV) depend on the regulation of the vagal nerve on the sinoatrial (SA) node and the atrioventricular node (AV node). Green lines represent the participation of sensory neurons.

The stress response is mediated by the ANS and the balance between the SNS and PNS [1]. Immediately after the perception of a stressor or a stimulus that threatens the animal’s homeostasis [4,17,18], several physiological, endocrine, and metabolic changes are triggered to restore homeostasis. The SNS modulates the activity of the cardiovascular, gastrointestinal, respiratory, renal, and endocrine systems, among others [19]. Its activation increases the HR, cardiac output, and blood flow to key organs (Figure 1) [1,20]. Increased body temperature (BT) can also be observed as well as changes in the pupillary diameter as the integrated fight–flight response [21,22,23]. 

The sympathetic response is mediated by the locus coeruleus (LC) and the noradrenergic system. LC increases the release of NE to increase arousal and alertness [19,24]. Additionally, SAM releases the catecholamines epinephrine (E) and NE [1,20]. For example, when rats are exposed to immobilization as the stressor, Pacak et al. [25] reported an increase in the synthesis, release, and metabolism of NE in the paraventricular nucleus, the central nucleus of the amygdala, the bed nucleus of the stria terminalis (BNST), and the hypothalamus. Similarly, mice experiencing five days of restraint stress increased their NE release in the BNST by 3.3 to 5-fold [26]. In the case of pigs, Bozzo et al. [27] studied the activation patterns of the SAM and HPA axes during transport stress to determine the effect that the journey duration and loading position have on the endocrine profile of animals (plasma E, NE, and cortisol (CORT)). The authors found that pigs undergoing long journeys (11 h long) had significantly higher concentrations of stress hormones (E: 19,414 ng/L; NE: 17,469 ng/L; CORT: 139 nmol/L) than those transported for three hours (E: 9232 ng/L; NE: 6227 ng/L; CORT: 94 nmol/L), which is associated with a higher level of stress. In cattle, Rodríguez et al. [28] reported that Holstein–Friesian cows with lameness (mobility score of 3) had higher NE concentrations (901.28 ± 33.66 pg/mL) than those with mobility scores of 0 (655.83 ± 24.09 pg/mL) as a result of pain (an event that is also considered a stressor). 

One of the main noradrenergic responses to psychogenic stressors is tachycardia due to increased plasma NE concentrations. Both E and NE can bind to adrenoreceptors (α1, α2, β1, β2, and β3) present in the endothelial and cardiac tissue, modifying the cardiovascular function [1,29,30]. In particular, β1 is mainly found in the heart and it regulates HR, contractility, and plasma renin release, being directly involved in HR increases; however, during stress reactions, β2 also contributes to tachycardia [31,32]. This has been observed in dogs during veterinary examination, an event that is frequently associated with other negative responses such as tachypnea, hypertension, mydriasis, hyperthermia, and increased muscular tension [33]. This was studied in 30 dogs of different breeds and ages during physical examination. When comparing average basal values in the waiting room of 97.7 ± 19.6 beats per minute (bpm) against 123.21 ± 21.2 bpm in the consulting room, a significant increase in the heart rate (HR) was observed, particularly when patted by the examiner (138.0 ± 25.2 bpm) and during a simulated vaccination (133.8 ± 28.7 bpm) [34]. 

Another event that can increase HR in animals is thermal stress since increases in HR and respiratory rate (RR) occur to promote heat dissipation, as reported in Boer goats exposed to high environmental temperatures (26, 29, and 33 °C) [35]. In this study, when animals were under air temperatures of 33 °C, HR and RR increased by 50% and pupillary diameter significantly increased by 37%. Moreover, a strong positive correlation between the increase in physiological parameters and pupillary diameter was found (r = 0.80) [35]. In cattle, Holstein bull calves maintained in non-shaded areas with an average temperature of 44.3 °C had higher HR, particularly in the afternoon (22.3 ± 1.4 bpm). Furthermore, RR also increased in non-shaded bulls [36]. 

Tachypnea increases the oxygen supply required by the high oxygen demand of muscle tissue during stressful events [1]. In the case of sporting animals such as dogs and horses, a higher RR increases the availability of arterial O_2_, which is facilitated by the increase of cardiac activity and, consequently, the volume of blood ejected [37,38]. Martins-Pinge [39] mentions that the sympathetic branch of the ANS plays a key role in modulating the cardiorespiratory system during aerobic exercise, adjusting its function to the animal’s requirements. Therefore, the ANS is vital to achieving stability in adverse conditions but also under physiological conditions of high demand such as exercise.

Another autonomous response that can be assessed in animals under stress is the changes in the pupil diameter of the species. Pupils can contract or dilate according to the level of activity of the ANS [40]. Noradrenergic and cholinergic circuits control the pupil diameter and size by direct innervation to the sphincter and the dilator muscle [41,42,43]. In this sense, pupillary constriction is modulated by postganglionary parasympathetic fibers through short ciliary nerves that innervate the pupillary sphincter diameter [44]. In contrast, pupil dilation is associated with sympathetic predominance and innervation to the iris dilator muscle and the release of NE after the activation of the ANS [45,46,47]. Due to the connection between the NE and pupil control, pupil diameter could serve as an indirect method to evaluate the activation of the ANS during stressful or painful events in animals [46,47].

Machado et al. [48] evaluated the pupil area, BT, and CORT concentrations in 360 piglets subjected to long-distance transport (75 km). The pupil area increased by 19.3%, along with a significant increase in CORT by 2.37 ng/mL and BT by 1.24 °C. Additionally, high positive correlations between pupil area, CORT, and BT were reported (r = 0.92, 0.90, and 0.82, respectively). In cats, the reaction of the animals to restraint during a physical examination was analyzed using the pupil dilation ratio, RR, and behavioral responses. In comparison with animals receiving passive restraint, cats subjected to full body restraint resulted in a larger pupil dilation ratio (approximately 0.7 vs. 0.45) and presented higher RR (29 vs. 18 bpm) and lip licking (2.3 vs. 1.5 licks/min). This possibly reaffirms the idea that activation of the ANS helps animals to compensate for changes during stressors and in this way could recover their stability [49]. 

As shown above, CORT release is also associated with the activation of the ANS because the response to stressors is mediated by the simultaneous activation of the SNS, SAM, and HPA axes [19,50,51]. Several studies have reported increases in CORT levels when animals are exposed to stressors such as heat stress. In this sense, Kim et al. [52] found that Korean native beef calves exposed to high THI levels (87.72) had serum CORT increases (17.1 ng/mL) as well as higher HR (73 bpm) and RT (39.9 °C) than calves under a THI of 70.01 (4.8 ng/mL, 60.3 bpm, and 38.9 °C, respectively). Another example is during equestrian events where horses can be exposed to potential stressors. Olvera-Maneau et al. [53] studied this response in pure-breed Menorca stallions and the effect that these festivals have on the salivary CORT concentration of animals. The authors found that CORT significantly increased to up to 3.8 nmol/L during the event, suggesting that it represents an acute stressor for the animals. Therefore, assessment of autonomic activity can be an additional parameter to monitor health and the current state of the animals. 

## 3. Heart Rate Variability and Its Association with Autonomic Activity

During the perception of a stressor, the activation of the ANS modifies the cardiovascular activity to mobilize energy resources [54]. As described by von Borell et al. [55], the cardiovascular system is modulated by the ANS and its two branches: sympathetic and parasympathetic, where both the SNS and PNS can increase or decrease HR, respectively [1,56]. A predominant vagal (or parasympathetic) tone regulates cardiac activity and maintains the beat-to-beat interval. A healthy heartbeat is characterized by irregular time intervals between consecutive beats. This variability is consequent to the rhythmic oscillation of the regulatory components of cardiac activity to maintain cardiovascular homeostasis. When fluctuations in vagal tone are present, these can be related to variations in the vagal nuclei activity, which are mediated by the baroreceptors. Thus, HR variability (HRV) is affected by the responsiveness to O_2_ and CO_2_ levels in baroreceptors during the respiratory cycle [57,58].

This could be related to Ille et al.’s [59] findings in 16 sport horses divided into two groups according to their level of exercise experience. Salivary CORT, HR, HRV, and standard deviation of the successive R-R interval were assessed. The authors observed that the HR in both groups increased by 50 bpm after the exercise; however, HRV and standard deviation of the R-R interval decreased by 20 ms. The decrease in both parameters is indicative of increased sympathetic tone that shortens the interval between R-R complexes and results in a greater number of heartbeats. 

**Figure 2:**
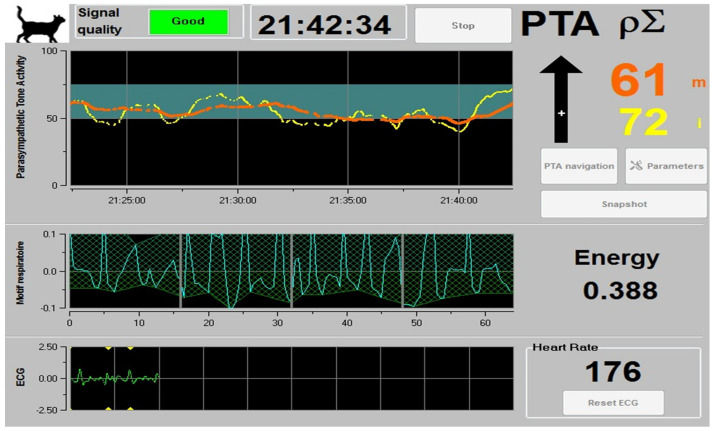
Screenshot of the parasympathetic tone index (PTA) monitor evaluating a 3-year-old shorthair domestic cat. The feline patient was premedicated orally with 5 mg/kg of pregabalin before undergoing an ovary hysterectomy. It can be observed that the PTA index is greater than 60%. This indicates a predominant parasympathetic tone due to the anxiolytic effect of pregabalin, which reduces stress during transport and routine consultation. Orange lines and number represent real time values; yellow lines and numbers represent 2-min average values. Picture taken by the authors.

The presence of these oscillations can reflect the level of ANS activity—whether SNS or PNS—by assessing the oscillation changes of HRV [60]. During potentially stressful events such as pain or nociception, the shortening between R-R complexes occurs due to tachycardia. Additionally, changes in the respiratory pattern stimulate baroreceptors and shift to a predominant sympathetic tone [61,62,63,64]. Currently, a numerical index to assess the level of parasympathetic tone through HRV has been developed, known as the Parasympathetic Tone Activity (PTA) index [64,65,66]. The PTA index is calculated using the following formula: PTA = (100 + [α + aucMIN + β]/12.8) + 100/161, where the value for α is 5.1 and β is 1.2, predetermined values to maintain the coherence of the respiratory influence on the series of RR intervals and the quantitative value of PTA. Thus, 100/161, 100/171, and 100/163 are predetermined coefficients for dogs, cats, and horses, respectively [67,68]. The PTA index uses a 0–100 scale, where values close to 100 indicate a relative parasympathetic tone, while those close to 0 represent diminished parasympathetic activity or a sympathetic predominance [69]. Figure 2 shows a PTA index greater than 60% in a feline patient due to the effect of the administration of anxiolytics.

Some studies in both dogs and horses have suggested that PTA can help to recognize the level of pain or nociception in animals during surgical procedures and assess the analgesic efficacy of drugs [67,70]. For example, Leitäo et al. [71] applied this index in healthy and anesthetized Large White pigs receiving a noxious stimulus and two types of analgesics (ketorolac and ketorolac/tramadol). Pigs receiving both analgesic treatments had a PTA mean value that was 30 units lower than pigs to whom drugs were not administered. Moreover, the HR of animals without analgesia was 45 bpm higher than medicated subjects, which reflects the ANS activation due to the painful stimulus. Similarly, Hernández-Ávalos et al. [72] used the PTA index as a method to assess the level of postoperative analgesia in 30 healthy bitches undergoing ovariohysterectomy. Although no variation was observed in the initial and postoperative value of the PTA index, a clinical relationship was observed between an index value of 40–49 with a score below 10 on the University of Melbourne Pain Scale, showing a sensitivity and specificity of 40% and 98.46%, respectively. Thus, through the evaluation of HRV due to its regulation by the ANS, it is possible to determine the predominant tone during adverse states such as pain or stress. It is therefore possible that the evaluation of the same is likely to determine the degree of analgesia during surgical procedures.

In other studies, the PTA index has been used as a method to predict changes in arterial pressure following the idea that changes in the sympathetic/parasympathetic balance alter cardiovascular function. In anesthetized horses, Mansour et al. [68] determined that values in the PTA decreased five minutes before hypotension, recognizing this event with a sensitivity and specificity of 62.5% and 94.6%, respectively. Similarly, intraoperative nociception and cardiovascular changes have been evaluated in Beagle dogs [73]. In these animals, it was reported that the PTA index was able to detect nociception even in the absence of cardiovascular changes, reaching PTA values as low as 25 ± 15. In the same species, Mansour et al. [74] compared the effect of three anesthetic protocols (morphine, morphine + medetomidine, and morphine + acepromazine) through the PTA index to identify intraoperative hemodynamic reactions of animals. During cutaneous incision, PTA values significantly decreased (range −21 to −23%) while increases in median arterial pressure (MAP) (between +27–28%) and HR (between +3.5–10.8%) were registered, except on animals receiving morphine + medetomidine. In contrast, Ruíz-López et al. [75] reported that PTA values below 51 were associated with hemodynamic response in bitches undergoing ovariectomy (increases in MAP but not in HR) but the sensitivity and specificity of PTA were considered poor (69 and 52%) to predict these changes. Therefore, further studies are required to establish the usefulness of PTA in assessing the cardiovascular activity of the ANS when no significant changes are observed in the HR, as described by Lima et al. [76] in cats undergoing ovariectomy. In this study, PTA was effective in detecting nociception when increases in HR above 20% reflect the activation of the SNS. 

## 4. Stress-Mediated Thermal Response and Its Assessment through Infrared Thermography

A phenomenon known as stress-induced hyperthermia occurs when the BT of animals increases after the perception of a stressor [56,77]. When psychological stress is perceived by the amygdala, projections to the dorsomedial hypothalamus and the preoptic area of the hypothalamus (POA) modulate the mechanisms to increase core temperature [78]. Through the activation of the hypothalamic–medullary–sympathetic axis, catecholamines are released, increasing cardiovascular activity [79,80]. Neurons located in the rostral raphe pallidus nucleus and the intermediolateral cell column promote thermogenesis in brown adipose tissue and cutaneous vasoconstriction [78,81,82]. Increases in NE can be accompanied by increases in adrenocorticotropic hormone, CORT, and glucose, which are known as stress hormones that increase the activity of the cardiovascular system and promote heat production [83,84,85]. 

Increases in BT also involve peripheral thermoregulation mechanisms mediated by the POA because mammals regulate their BT by balancing the amount of metabolically produced heat and the one that is exchanged with the environment [86]. During the perception of a stressor—and as a response to SIH—the diameter of cutaneous blood vessels changes, promoting vasodilation to increase blood flow and heat dissipation [12]. Estimation of these vasomotor changes can be assessed through IRT in different body regions called thermal windows [13,14]. Thermal windows are characterized by regions with poor insulation or glabrous skin, rich vascular beds, and arteriovenous anastomoses that facilitate heat transfer [86] and might be a tool that indirectly assesses the activity of the SNS [15,16].

**Figure 3:**
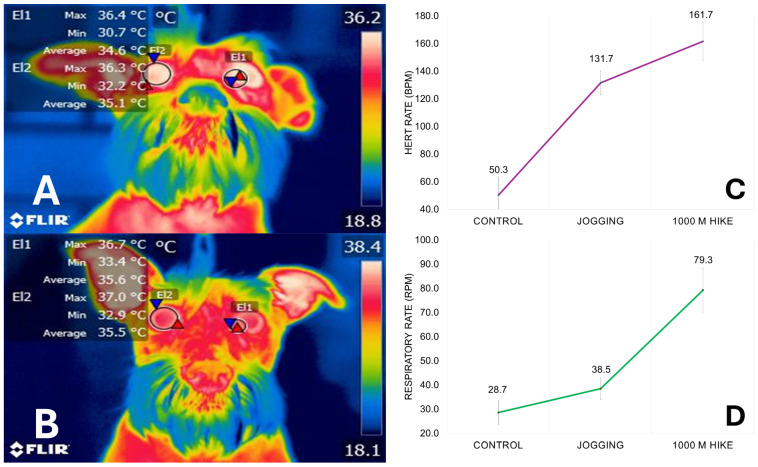
Surface thermal response of dogs during exercise. (**A**) Control measurements of a Schnauzer dog. The maximum, minimum, and average temperature of the lacrimal caruncle (El1) and the ocular surface (El2) can be observed, being 36.4, 30.7, and 34.6 °C. (**B**) Surface temperature after jogging. When comparing the average values of the lacrimal caruncle and ocular temperature, a difference of 1 °C and 0.4 °C, respectively, was reported, showing the increase in these thermal windows due to peripheral vasodilation. Graphs (**C**,**D**) show the increase in the heart rate and respiratory rate of the dogs included in the pilot study. Red triangle: maximum temperatures; blue triangle: minimum temperatures. Images taken with a FLIR E60 thermal camera, lens FOL 18 mm, IR resolution of 240 × 180, emissivity of 0.95, distance from object 30 cm.

Ocular surface temperature has been correlated with autonomic modulation and glucocorticoid concentrations, suggesting it as a biomarker for indirect assessment of ANS activity [56]. In particular, the lacrimal caruncle is considered a region that reflects the autonomic activity [87,88,89]. In this sense, Shu et al. [90] determined the correlation of the ocular temperature of dairy cows under heat stress assessed in five points: medial canthus, lateral canthus, eyeball, whole eye, and lacrimal sac. The highest correlation between RT and RR was found in the lacrimal sac (up to 0.60), concluding that this region should be considered as an adequate predictor of heat stress in animals. According to Mota Rojas et al. [8], the blood flow of the lacrimal caruncle is provided by the orbital artery and its branches (supraorbital and infraorbital artery) [91]. The suborbital artery receives sympathetic innervation from the facial nerve [92]. Therefore, changes in the surface temperature of this region can reflect sympathetic-mediated responses on skin blood flow, as shown in Figure 3. A pilot study made by the present authors evaluated the response of six dogs during an exercise test, where the average temperature of the lacrimal caruncle and the ocular surface was compared before and after exercise (jogging and 1000 m hiking). In this study, increases in the temperature of both thermal windows were recorded (of up to 1 °C) after 1000 m jogging. Additionally, the HR and RR of dogs also increased from control measurements to the end of the exercise, reaching an increase of up to 111.4 bpm and 50.6 rpm, respectively. This response is associated with a predominant sympathetic tone that increases BT and triggers cutaneous vasodilation, as well as tachycardia and tachypnea to compensate for the increased cardiac and muscular activity.

**Figure 4:**
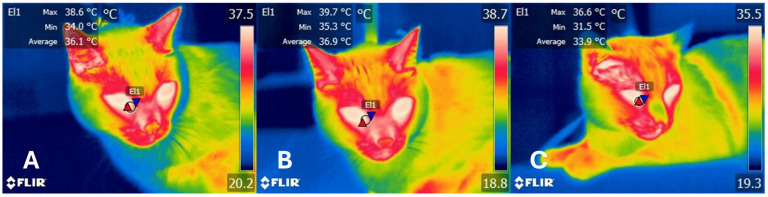
Thermal imaging to assess the autonomic response of cats exposed to different stressors. (**A**) Basal measurements in a male domestic shorthair cat. When the cat was not exposed to any stressor, a maximum temperature of the lacrimal caruncle (El1) of 38.6 °C, a minimum of 34.0 °C, and an average value of 36.1 °C was recorded. (**B**) After the exposure to an unfamiliar dog, an increase in the three temperatures was observed, registering increases of 1.1 °C, 1.3 °C, and 0.8 °C, respectively. (**C**) After the use of a cat appeasing pheromone, it can be observed that the surface temperature decreased when compared to B values (average of 3 °C). Furthermore, the cat pheromones caused lower temperatures than basal values (as low as −2.5 °C) and this might be related to the calming effect of the pheromones due to a predominant parasympathetic tone. Images taken with a FLIR E60 thermal camera, lens FOL 18 mm, IR resolution of 240 × 180, emissivity of 0.95, distance from object 30 cm. Red triangles: maximum temperatures; blue triangles: minimum temperatures.

Arfuso et al. [93] used IRT to evaluate the response of sheep to acute stress during a shearing procedure. By collecting blood samples, RT, and ocular surface temperature, the authors found that RT and the temperature of the medial canthus after shearing significantly increased by 0.5 °C. Simultaneously, increases in blood concentration of CORT were registered, values that were positively correlated with surface and rectal temperature (r = 0.70). Similarly, ocular surface temperature was assessed in thirty bull calves undergoing surgical castration with local anesthesia and sham handling control surgical castration. In the control animals, increases in HR (by 15 bpm) and eye temperature (by 0.47 °C followed by a decrease of 1 °C) were found after the castration procedure. This coincided with the significant increase of 7.3 ng/mL of CORT after castration, changes that are after autonomic activity and the surface thermal response [2]. Figure 4 shows the preliminary results obtained by the present authors when evaluating ocular thermal response of cats exposed to a stressor (dog) and to the effect of a cat appeasing pheromone, stimulus known to activate the sympathetic and parasympathetic system, respectively. 

In calves subjected to disbudding, the maximum eye temperature measurements (located at the lacrimal caruncle) detected changes between animals receiving local anesthetics and those that did not receive analgesics. A sudden drop in temperature (−0.27 °C) followed by an increase in eye temperature of up to 0.60 °C was recorded in calves without local anesthetics, an alteration related to an acute sympathetic response to pain [94]. During heat stress Chikkagoudara et al. [95] reported that the eye temperature of young buffalo bulls increased significantly when the temperature humidity index increased (up to 37.41 °C when the index was 48.34), showing that surface temperature can be used as an indicator of stress. 

Although these studies show that IRT can indirectly assess ANS activity, Sutherland et al. [96] mentions that HRV is a more sensitive method than IRT, as observed in their study evaluating the effect of E infusion on the temperature of the lacrimal gland and HRV in 20 Romney ewes. The authors found that the infusion of epinephrine increased the ocular temperature (by 0.3 °C). However, increases in HR and changes in HRV were considered more sensitive to measure ANS activity. Thus, the cardiovascular changes that occur during stressful events can be detected through IRT. Using thermal imaging as a complementary tool to evaluate the response of animals to stress might help to recognize the predominant tone of the ANS.

## 5. Perspectives about Thermal Imagining

Due to the low sensitivity of IRT in comparison to other techniques, it has been suggested that further studies should focus on the difference and comparison of the different thermal windows or anatomical regions [7,89]. Likewise, since pain perception is highly modulated by the SNS, IRT could be a useful tool to assess the analgesic effect of certain drugs, as observed in dogs under controlled procedures [97]. Moreover, Bergamasco et al. [98] reported that eye surface temperature is mediated by the ANS but the response can differ according to the age of the animal in calves undergoing unmitigated castration. Therefore, considering the age of the animals might be needed in future studies. 

Currently, laser Dopler flowmetry is one of the main methods to non-invasively measure skin blood flow and recognize changes in microvascular perfusion [99]. In humans, several studies have applied the combined use of IRT with Doppler flowmetry to assess the angiospatic dysfunction of peripheral blood flow [100]. Authors such as Merla et al. [101] established that combining laser Doppler, thermal imagining, and bio-heat transfer modeling helps to discriminate healthy patients from those with impaired blood flow, but that thermal imaging is considered a faster technique for real-time detection of cutaneous perfusion alterations. 

Other studies propose a thermal-associated pain index using skin conductance, HRV, and peripheral perfusion to optimize pain assessment in patients. Although this index has only been studied in humans, it has shown a sensitivity above 70% to detect pain, suggesting that it could be adapted to veterinary medicine. Furthermore, estimation of the HRV using facial thermal imaging is proposed as a contactless method to identify cardiovascular changes affecting HRV and HR, showing strong positive correlations (r = 0.7 and 0.67, respectively). Using IRT in combination with other techniques is suggested as a reliable protocol to recognize the activation of the ANS. 

We used parallel or series measurement to increase the sensitivity and specificity of IRT measurements, and even compared the predictive value of thermal imaging to assess activation of the ANS [102]. For example, in humans, IRT as a diagnostic tool has a positive predictive value of 72.2, a negative predictive value of 93.3, and an overall sensitivity, specificity, and accuracy of 87.1, 87.2, and 89.7, respectively [103]. However, most studies have been performed in humans and a large variation in sensitivity and specificity due to operating characteristics has been reported. Therefore, its application in veterinary medicine, together with other tools, needs to be evaluated. Moreover, automatic systems adopting infrared thermography could be another strategy that could help recognize shifts in the activity of the ANS that might be related to stress or pain [104,105]. This could be useful in clinical or farm settings where a prompt recognition of autonomic alterations might prevent further alterations in animals. 

## 6. Conclusions

Changes in the skin blood flow—vasoconstriction or vasodilation– due to stress are mediated by the ANS. Oscillations in skin blood flow modify the skin temperature, a parameter that can be assessed through IRT. During the perception of a stressor, the sympathetic predominance causes an increase in RT as well as in the surface temperature of animals. In particular, thermal imaging focused on the lacrimal caruncle is considered an adequate predictor to evaluate the activity of the ANS due to the predominant sympathetic innervation in this region. However, since IRT interpretation depends on the thermal window of animals, thermal imagining is recommended as a complementary method that should be used together with other sensitive and specific techniques (e.g., laser Doppler) to determine the autonomic control of skin blood flow. 

## Data Availability

Data sharing not applicable.
